# Consumption of apples is associated with a better diet quality and reduced risk of obesity in children: National Health and Nutrition Examination Survey (NHANES) 2003–2010

**DOI:** 10.1186/s12937-015-0040-1

**Published:** 2015-05-14

**Authors:** Carol E. O’Neil, Theresa A. Nicklas, Victor L. Fulgoni

**Affiliations:** 1 Louisiana State University Agricultural Center, 261 Knapp Hall, Baton Rouge, LA 70803 USA; 2 Department of Pediatrics, USDA/ARS Children’s Nutrition Research Center, Baylor College of Medicine, Houston, TX 77030 USA; 3 Nutrition Impact, LLC, Battle Creek, MI 49014 USA

**Keywords:** NHANES, Apple, Apple juice, Apple sauce, Children, Fruit, Weight, Diet quality

## Abstract

**Background:**

Most children do not meet the recommendation for fruit consumption. Apples are the second most commonly consumed fruit in the US; however, no studies have examined the association of total apple products, apples, apple sauce, and 100 % apple juice consumption on diet quality and weight/adiposity in children.

**Methods:**

The purpose of this study was to examine the association between various apple consumption forms with diet quality and weight/adiposity in a nationally representative sample of children. Participants were children 2–18 years of age (*N* = 13,339) from the National Health and Nutrition Examination Survey 2003–2010. Intake was determined using a single interview administered 24-h diet recall. Apple product consumption was determined using the cycle-appropriate USDA Food and Nutrient Database for Dietary Studies food codes. Total diet quality and component scores were determined using the Healthy Eating Index-2010 (HEI). Anthropometrics were determined using standard methods. Covariate adjusted linear and logistic regressions were used to compare apple product consumers with non-consumers; sample weights were used. Probability was set at <0.01.

**Results:**

Approximately 26 % of the population (*n* = 3,482) consumed some form of apple products. Consumers of apple products, whole apples, apple sauce, and 100 % apple juice had higher HEI scores than non-consumers: 50.4 ± 0.4 v 41.9 ± 0.3, 52.5 ± 0.5 v 42.7 ± 0.3, 52.1 ± 0.8 v 47.2 ± 0.4, and 51.4 ± 0.6 v 46.5 ± 0.4, respectively. Apple products and whole apple consumers had lower BMI z-scores than non-consumers: 0.4 ± 0.04 v 0.5 ± 0.03 and 0.3 ± 0.1 v 0.5 ± 0.02, respectively. Apple products and whole apple consumers were 25 % (0.59–0.95 99^th^ CI) and 30 % (0.52–0.95 99^th^ CI), respectively, were less likely to be obese than non-consumers.

**Conclusions:**

Consumption of any form of apples contributed to the fruit recommendation of children and improved diet quality. Apples should be included in the diets of children as a component of an overall healthy diet.

## Introduction

Fruit, defined by the Dietary Guidelines for Americans (DGA) [1] as a nutrient-dense food, has also been recognized as part of a healthy eating pattern [1]. Eating nutrient-dense foods, such as fruit helps Americans balance nutrient needs within their energy needs. The recommendation for fruit is age, gender, and physical activity dependent, and for children ranges from 1 cup equivalent for children 2–3 years of age (years) to 2 cup equivalents for males 14–18 years [2]. Fresh, frozen, canned, or dried fruit can be used to meet the fruit recommendation, as can 100 % fruit juice [2, 3]. Approximately 35 % to 50 % of total fruit intake by children 9 to 18 years [4] comes from 100 % fruit juice, which makes a positive contribution to overall diet quality, and has been associated with increased intake of whole fruit [5–7]. However, most children fail to meet the fruit recommendation [8–14].

Eating a diet rich in fruit, as part of an overall healthy diet may reduce the risk of cardiovascular disease [15–18], type 2 diabetes [18–20], and some types of cancer [18, 21]. According to the DGA [1], eating fruit, which is a relatively low-energy food, in place of higher energy foods may help lower overall energy intake; however, the effect of fruit consumption on weight or weight loss is controversial [18, 22–24]. Fruit provides a wide array of nutrients, including nutrients of public health concern [1], such as dietary fiber and potassium, as well as other shortfall nutrients, like vitamins A and C and folate [25]. Many of the health benefits seen may be due to these nutrients or to the phytochemicals found in fruit [26].

Apples (*Malus domestica*) are the second most commonly consumed fruit in the United States (US) [27], with 65 % of the apple crop consumed as fresh fruit and 35 % as processed apple products (e.g. apple sauce or apple juice) [28]. One medium raw apple (182 g), with skin, provides approximately 95 kcals, 19 g total sugars, 4 g dietary fiber (22 % of the Daily Value [DV]), and 195 mg of potassium (6 % DV). In addition, raw apples contain virtually no total fat, saturated fatty acids, or sodium; and they have no cholesterol. Processed apple products have a slightly different nutrient profile than raw apples. For example apple sauce may or may not have added sugars and ½ cup (122 g) has only 1.3 g dietary fiber and ½ cup of 100 % fruit juice (124 g) has 0.2 g dietary fiber [25]. These processed apple products still count toward the fruit recommendation [2]. Apples are also especially rich in phenolics, especially hydroxycinnamic acid derivatives and flavonoids [27].

No studies have examined the association of apple product consumption and diet quality or weight/adiposity parameters in children. The purpose of this study was to examine the association between apple/apple product consumption with diet quality and weight/adiposity parameters in a nationally representative sample of children using the National Health and Nutrition Examination Survey (NHANES) 2003–2010 data.

## Subjects and methods

### The NHANES

The NHANES is a continual program designed to collect data that can be used to assess the health and nutritional status of free-living children and adults in the US. One of major objectives of the NHANES is to provide the data for investigators to be able to examine the relationship between diet, nutrition, and health [29]. The survey is unique in that it collects data from interviews, dietary intake, and physical examinations. Details regarding the survey design, content, operations, procedures, and participation rates are available online [29–32].

### Study population and dietary intake

The study population consisted of children 2–18 years (*N* = 13,339) who participated in the 2003–2004, 2005–2006, 2007–2008, 2009–2010 NHANES. Intake data were obtained from What We Eat in America which were collected during an in-person automated multiple-pass 24-h dietary recall interview and a telephone 24-h dietary recall conducted three to ten days later [33, 34]. For these studies, only the first day of data collection was used. Detailed descriptions of the dietary interview methods are provided in the NHANES Dietary Interviewers Procedure Manuals [35, 36]. Briefly, proxies, usually parents, provided the 24-h dietary recalls of children 2–5 years; children 6–11 years were assisted by a proxy; older children provided their own recalls. Recall data deemed unreliable by the USDA Food Surveys Research Group (*n* = 275), pregnant and lactating females (*n* = 83), and those children consuming breast milk (*n* = 10) were excluded from the analyses. This left a final analytical sample of 13,339. The NHANES has stringent protocols and procedures that ensure confidentiality and protect individual participants from identification using federal laws [37] and additional Institutional Review Board approval for these secondary analyses was not required [38].

### Determination of apple product consumption

Apple/apple product consumption was determined form the 24-h dietary recall by using the cycle-appropriate United States Department of Agriculture food codes [39] for: 1) whole apples; 2) apple sauce (which includes cooked apples); 3) 100 % apple juice; and, 4) total apples, which included all food codes from the three groups above.

### Healthy Eating Index (HEI-2010)

The HEI-2010 [40, 41] was used to determine diet quality as specified by the 2010 DGA [1]. For the HEI-2010 a total score is determined, as are 12 component scores. Of these, nine: total fruit, whole fruit, total vegetables, greens and beans, whole grains, dairy, total protein foods, seafood and plant proteins, and fatty acids measure adequacy; and a higher score is better. Three of the component scores: refined grains, sodium, and empty calories, measure moderation; and higher scores indicate lower consumption. The SAS code used to calculate HEI-2010 scores was downloaded from the Center for Nutrition Policy and Promotion website [42].

### Anthropometric and physiological measures

Height, weight, and waist circumference were obtained according to NHANES protocols [43]. Body mass index (BMI) was calculated as kg/m^2^ [44]. The SAS program of the Centers for Disease Control and Prevention was used to determine BMI z-score and placement of children on the 2000 growth charts [45]. Children with a BMI ≥ the 85^th^ and <95^th^ were considered overweight; and children ≥ the 95th percentile were considered obese [46]. Children with a BMI ≥ 85^th^ percentile were considered overweight or obese.

### Statistical analyses

Sampling weights and the primary sampling units and strata information, as provided by NHANES [47], were included in all analyses using SUDAAN v11.0 (Research Triangle Institute; Raleigh, NC). Least-square means (and the standard errors of the least-square means) were calculated using PROC REGRESS of SUDAAN. Linear regression was used to determine differences in total and sub-component HEI-2010 scores between apple, apple sauce, apple juice, and total apple consumers and non-consumers, as well as differences in anthropometric measures among these consumers and non-consumers. Logistic regression was used to determine if apple, apple sauce, apple juice, and total apple consumers had a lower odds ratio (OR) of being overweight, obese, or overweight or obese. For all linear and logistic regressions, covariates were: age (continuous variable), gender (discrete variable), and ethnicity (discrete variable), all of which were determined from the sample person questionnaire [31]; poverty index ratio (<1.25, 1.25–3.24, >3.25), which was obtained from the NHANES family questionnaire [48]; and physical activity level (sedentary, moderate, and vigorous), which was also obtained from the sample person questionnaire [31, 49]. All covariates were self-reported. A p value of <0.01 was considered significant to reduce the likelihood of making a type one error.

## Results

### Apple consumption and demographic characteristics

Approximately 26 % of the population (*n* = 3,482) consumed some form of apple products; 14 % (*n* = 1,891) consumed whole apple; 5 % (*n* = 332) consumed apple sauce, and ~12 % (*n* = 1,714) consumed 100 % apple juice. Some children consumed more than one apple product in one day; therefore, there was some overlap in the study population. Among consumers, mean intake of any apple products was 222.2 ± 3.9 g, whole apple was 143 ± 3.8 g (~1 cup equivalent), apple sauce was 129.8 ± 5.7 g (~1/2 cup equivalent), and apple juice was 9.6 ± 0.24 fluid ounces (272.5 ± 6.7 g; 1.2 cup equivalents). Total apple product consumers, whole apples, apple sauce, or apple juice were more likely to be younger and less likely to be current smokers than non-consumers (Table 1). There were also racial/ethnic differences among consumers of whole apples and apple sauce, with fewer non-Hispanic blacks and more Mexican-Americans consuming whole apples and fewer Mexican-Americans consuming apple sauce. Apple sauce consumers were less likely to be sedentary and more likely to be moderately physically active than non-consumers (Table 1).

**Table 1:** Demographic characteristics of children 2–18 years (N = 13,339) participating in NHANES 2003–2010 by total apple and apple product consumption

	Total apple products			Whole apples			Apple sauce			Apple juice		
Variable	Consumers (*n* = 3,482) LSM ± SE	Non-Consumers (*n* = 9,857) LSM ± SE	P-value	Consumers (*n* = 1,891) LSM ± SE	Non-Consumers (*n* = 11,448) LSM ± SE	P - value	Consumers (*n* = 332) LSM ± SE	Non-Consumers (*n* = 13,007) LSM ± SE	P-value	Consumers (*n* = 1,714) LSM ± SE	Non-Consumers (*n* = 11,625) LSM ± SE	P-value
Gender (%)												
Female	49.9 ± 1.4	49.0 ± 0.8	0.584	50.4 ± 1.8	49.0 ± 0.8	0.466	49.5 ± 3.2	49.2 ± 0.8	0.930	49.9 ± 2.1	49.1 ± 0.8	0.732
Ethnicity (%)												
NHW	58.3 ± 2.0	61.0 ± 2.0	0.334	57.1 ± 2.3	60.8 ± 2.0	0.220	76.7 ± 3.2	59.7 ± 1.9	**<0.001**	55.6 ± 2.6	60.9 ± 1.9	0.105
NHB	13.0 ± 1.1	14.9 ± 1.1	0.227	10.6 ± 1.1	15.0 ± 1.1	**0.004**	12.4 ± 2.0	14.5 ± 1.0	0.367	15.4 ± 1.4	14.3 ± 1.0	0.508
MA	15.9 ± 1.3	12.4 ± 1.2	0.039	18.0 ± 1.5	12.5 ± 1.2	**0.004**	5.2 ± 1.3	13.6 ± 1.2	**<0.001**	16.1 ± 1.6	12.9 ± 1.1	0.104
Age (Years)	8.3 ± 0.2	10.7 ± 0.1	**<0.001**	9.1 ± 0.2	10.3 ± 0.1	**<0.001**	7.2 ± 0.3	10.2 ± 0.1	**<0.001**	7.2 ± 0.2	10.5 ± 0.1	**<0.001**
PIR	2.6 ± 0.1	2.5 ± 0.1	0.486	2.6 ± 0.1	2.5 ± 0.1	0.155	2.6 ± 0.2	2.5 ± 0.1	0.504	2.4 ± 0.1	2.5 ± 0.1	0.159
PA (%)												
Sedentary	13.0 ± 1.0	12.7 ± 0.6	0.839	12.4 ± 1.3	12.9 ± 0.5	0.722	8.2 ± 1.7	12.9 ± 0.5	**0.009**	16.0 ± 1.6	12.3 ± 0.5	0.032
Moderate	20.4 ± 1.1	19.8 ± 0.7	0.676	20.4 ± 0.7	17.6 ± 1.6	0.115	32.7 ± 3.7	19.6 ± 0.7	**<0.001**	20.8 ± 1.7	19.9 ± 0.7	0.590
Active	66.7 ± 1.3	67.5 ± 0.9	0.621	66.8 ± 0.8	70.0 ± 2.2	0.164	59.1 ± 3.7	67.5 ± 0.8	0.025	63.2 ± 1.9	67.8 ± 0.8	0.025
Current smoker (%)	2.5 ± 0.4	7.5 ± 0.5	**<0.001**	6.8 ± 0.4	2.3 ± 0.4	**<0.001**	1.3 ± 0.7	6.3 ± 0.4	**<0.001**	2.6 ± 0.7	6.7 ± 0.4	**<0.001**
Alcohol (g)	0.3 ± 0.1	0.6 ± 0.1	0.012	0.5 ± 0.1	0.3 ± 0.1	0.075	0.0 ± 0.0	0.5 ± 0.1	**<0.001**	0.2 ± 0.1	0.5 ± 0.1	0.016

Data Source: Children 2 to 18 years of age participating in the NHANES 2003–2010

Bolded values are significantly different *p* < 0.01; statistical differences were assessed using z-statistics

Abbreviations: LSM = Least Square Means; SE = Standard Error; NHW = Non-Hispanic White; NHB = Non-Hispanic Black; MA = Mexican American; PIR = poverty index ratio; PA = physical activity

### HEI-2010

Although all HEI-2010 scores were relatively low, consumers of any apple product had higher total HEI-2010 scores than non-consumers (Table 2). Total apple product consumers also had higher HEI-2010 component scores: total fruit, whole fruit, whole grains, sodium, and empty calories than non-consumers. It is important to remember that sodium, refined grains, and empty calories are reverse scored so a higher score indicates lower consumption. Whole apple consumers had higher component scores for total and whole fruit, whole grains, and seafood & plant protein, as well as empty calories. Apple sauce consumers also had higher component scores for total and whole fruit, whereas apple juice consumers had higher component scores for total fruit and empty calories.

**Table 2:** Association between consumption of apple products, apples, apple sauce, and apple juice and Healthy Eating Index-2010 total and component scores in children participating in NHANES 2003–2010 (N  = 13,339)

Healthy eating index-2010 & Components	Total apple products			Apples			Apple sauce			Apple juice		
	Consumers (*n* = 3,482) LSM ± SE	Non-Consumers (*n* = 9,857) LSM ± SE	P-value	Consumers (*n* = 1,891) LSM ± SE	Non-Consumers (*n* = 11,448) LSM ± SE	P-value	Consumers (*n* = 332) LSM ± SE	Non-Consumers (*n* = 13,007) LSM ± SE	P-value	Consumers (*n* = 1,714) LSM ± SE	Non-Consumers (*n* = 11,625) LSM ± SE	P-value
Total Score	50.4 ± 0.4	41.9 ± 0.3	**<0.001**	52.5 ± 0.5	42.7 ± 0.3	**<0.001**	52.1 ± 0.8	47.2 ± 0.4	**<0.001**	51.4 ± 0.6	46.5 ± 0.4	**<0.001**
Total Vegetables	2.2 ± 0.1	2.1 ± 0.03	0.519	2.3 ± 0.1	2.1 ± 0.03	0.108	1.7 ± 0.1	2.0 ± 0.03	0.055	2.0 ± 0.1	2.0 ± 0.04	0.730
Greens & Beans	0.7 ± 0.1	0.6 ± 0.03	0.052	0.8 ± 0.1	0.6 ± 0.02	0.021	0.4 ± 0.1	0.6 ± 0.03	0.070	0.6 ± 0.1	0.5 ± 0.03	0.162
Total Fruit	4.2 ± 0.04	1.9 ± 0.1	**<0.001**	4.4 ± 0.1	2.2 ± 0.04	**<0.001**	4.0 ± 0.1	3.1 ± 0.1	**<0.001**	4.4 ± 0.1	2.9 ± 0.1	**<0.001**
Whole Fruit	3.6 ± 0.1	1.5 ± 0.1	**<0.001**	4.7 ± 0.04	1.6 ± 0.04	**<0.001**	4.4 ± 0.1	2.5 ± 0.1	**<0.001**	2.8 ± 0.1	2.5 ± 0.1	0.071
Whole Grains	2.0 ± 0.1	1.7 ± 0.1	**<0.001**	2.1 ± 0.1	1.8 ± 0.1	0.002	2.5 ± 0.3	2.1 ± 0.1	0.102	2.2 ± 0.1	2.1 ± 0.1	0.457
Dairy	7.1 ± 0.1	6.8 ± 0.1	0.028	7.2 ± 0.1	6.9 ± 0.1	0.043	8.0 ± 0.3	7.7 ± 0.1	0.227	7.8 ± 0.2	7.7 ± 0.1	0.521
Total Protein Foods	3.4 ± 0.04	3.5 ± 0.03	0.032	3.5 ± 0.1	3.5 ± 0.03	0.451	3.3 ± 0.1	3.3 ± 0.03	0.608	3.3 ± 0.1	3.4 ± 0.04	0.362
Seafood & Plant Protein	1.4 ± 0.1	1.3 ± 0.03	0.113	1.6 ± 0.1	1.3 ± 0.03	0.001	1.3 ± 0.2	1.4 ± 0.1	0.699	1.3 ± 0.1	1.4 ± 0.1	0.230
Fatty Acid Ratio	3.8 ± 0.1	3.7 ± 0.1	0.466	3.9 ± 0.1	3.8 ± 0.1	0.377	3.3 ± 0.3	3.4 ± 0.1	0.787	3.5 ± 0.2	3.3 ± 0.1	0.281
Sodium	5.3 ± 0.1	4.9 ± 0.1	**<0.001**	5.3 ± 0.2	5.0 ± 0.1	0.019	6.1 ± 0.4	5.4 ± 0.1	0.091	5.7 ± 0.2	5.4 ± 0.1	0.072
Refined Grains	5.4 ± 0.1	5.1 ± 0.1	0.022	5.3 ± 0.2	5.2 ± 0.1	0.408	6.2 ± 0.4	5.7 ± 0.1	0.300	6.0 ± 0.2	5.6 ± 0.1	0.064
Empty Calories	11.2 ± 0.2	8.6 ± 0.1	**<0.001**	11.5 ± 0.3	8.9 ± 0.12	**<0.001**	11.1 ± 0.4	10.1 ± 0.2	0.025	11.7 ± 0.3	9.8 ± 0.2	**<0.001**

Data Source: Children 2 to 18 years of age participating in the NHANES 2003–2010

Bolded values are significantly different *p* < 0.01; statistical differences were assessed linear regression

Abbreviations: LSM-Least Square Means; SE-Standard Error

Covariates: Gender, ethnicity, age, Poverty Income Ratio (PIR 0–1.25, 1.25–3.4, ≥3.50), and Physical Activity Level (Sedentary, Moderate, Active)

### Weight and adiposity measures

Total apple product and apple consumers had lower mean BMI z-scores than non-consumers (Table 3). Total apple product consumers also had a lower prevalence of obesity and overweight or obesity than seen in non-consumers; whereas, whole apple consumers only had a lower prevalence of obesity than non-consumers. No differences in any measure of weight or adiposity were seen between apple sauce and apple juice consumers, when compared with non-consumers. Table 4 shows that children consuming total apple products or whole apples were 25 % and 30 %, respectively, less likely to be obese than non-consumers. No significant differences were seen when comparing the likelihood of overweight or obesity among apple sauce or apple juice consumers and non-consumers.

**Table 3:** Weight status^a^ apple products, apples, apple sauce, and apple juice of children participating in NHANES 2003–2010 (N = 13,339)

	Consumers	Non-Consumers	P-value
	LSM ± SE^b^	LSM ± SE	
**Total Apple Products**			
BMI z-score	0.4 ± 0.04	0.5 ± 0.03	**0.009**
Waist Circumference (cm)	68.2 ± 0.3	68.9 ± 0.3	0.066
% Overweight	14.1 ± 1.0	15.5 ± 0.6	0.233
% Obese	13.5 ± 0.9	16.9 ± 0.8	**0.004**
% Overweight or Obese	27.6 ± 1.5	32.4 ± 1.0	**0.007**
**Whole Apples**			
BMI z-score	0.3 ± 0.1	0.5 ± 0.02	**0.008**
Waist Circumference (cm)	68.1 ± 0.4	68.9 ± 0.3	0.105
% Overweight	14.5 ± 1.6	15.2 ± 0.6	0.695
% Obese	12.6 ± 1.1	16.6 ± 0.7	**0.003**
% Overweight or Obese	27.1 ± 2.0	31.8 ± 1.0	0.040
**Apple Sauce**			
BMI z-score	0.4 ± 0.1	0.5 ± 0.02	0.301
Waist Circumference (cm)	67.7 ± 0.6	68.8 ± 0.2	0.101
% Overweight	14.2 ± 2.4	15.1 ± 0.6	0.688
% Obese	11.2 ± 2.0	16.2 ± 0.7	0.018
% Overweight or Obese	25.4 ± 3.2	31.3 ± 1.0	0.078
**Apple Juice**			
BMI z-score	0.4 ± 0.1	0.5 ± 0.02	0.502
Waist Circumference (cm)	68.5 ± 0.4	68.8 ± 0.2	0.528
% Overweight	15.6 ± 1.6	15.0 ± 0.6	0.722
% Obese	13.8 ± 1.2	16.3 ± 0.7	0.074
% Overweight or Obese	29.4 ± 2.2	31.4 ± 1.0	0.407

^a^Adjusted for age, gender, ethnicity, poverty income ratio, physical activity level (sedentary, moderate, vigorous)

Bolded values are significantly different *p* < 0.01; statistical differences were assessed linear regression

^b^SE: Standard Error; LSM: Least Squares Mean

**Table 4:** Odds ratios of waist circumference and weight status by apple product consumption

	Consumers^a^			
	OR^b,c^	LCL	UCL	P-value
**Total Apple Products**				
Obese	0.75	0.59	0.95	**0.002**
Overweight	0.89	0.72	1.11	0.173
Overweight or Obese	0.79	0.66	0.94	**0.001**
**Apples**				
Obese	0.70	0.52	0.95	**0.003**
Overweight	0.95	0.68	0.32	0.681
Overweight or Obese	0.79	0.60	1.04	0.027
**Apple Sauce**				
Obese	0.62	0.32	1.18	0.056
Overweight	0.92	0.54	1.58	0.693
Overweight or Obese	0.73	0.45	1.19	0.098
**Apple Juice**				
Obese	0.80	0.58	1.10	0.068
Overweight	1.05	0.76	1.45	0.707
Overweight or Obese	0.90	0.69	1.19	0.331

^a^Non-consumers are the comparison group

^b^Adjusted for age, gender, ethnicity, poverty income ratio, physical activity level

Non-consumers are the comparative group

^b,c^OR: Odds ratio; LCL: Lower 99th percentile confidence limit; UCL: Upper 99th percentile confidence limit; statistical differences were determined using logistic regression

## Discussion

This is the first study, that we are aware of, that has shown that total apple product consumption, whole apples, and apple sauce and apple juice were associated with higher diet qualities than those seen in non-consumers of the same food groups. It is also the first study to show that total apple consumption and whole apple consumption is associated with a lower prevalence of obesity and a lower likelihood of obesity.

Recently, it was shown that more than three-quarters (77.1 %) of all children 2–19 years consumed any fruit on a given day [8]. There is a clear difference in age, with younger children 2–5 years consuming more than older children 12–19 years [8]. Fruit consumption among children 2–18 years appears to have increased during the time of this study (2003 to 2010) [9] from 0.55 in 2003–2004 to 0.62 in 2009–2010 cup equivalents/1,000 kcal (kcals) because of significant increases in whole fruit intake. Similar results have been shown for children 2–17 years [10]. Fruit juice consumption declined over this period, going from 0.31 to 0.22 cup equivalents/1,000 kcals [9]. Despite these encouraging numbers, fruit is still under-consumed by children [11–14].

Consumption of total apple products and whole apples clearly has a significant impact on overall diet quality, suggesting that fruit intake and in particular apple products, should be encouraged. This complements other studies that have shown that consumption of individual types of whole fruit, including grapefruit [50], mangos [51], pears [52], and avocados [53], and 100 % fruit juice [6, 54] was associated with a higher HEI score than non-consumers. In this study, total apple products, apples, and apple sauce were all associated with intakes of greater intakes of total and whole fruit, whereas, consumption of 100 % fruit juice was not. This differs from other studies that have examined the diet quality of 100 % fruit juice consumers [6, 54], perhaps because of the number of 100 % apple juice consumers was relatively small compared with the total number of children consuming 100 % of any type of fruit juice. In addition, the higher diet quality for consumers of all apple products was also driven by higher component scores for whole grains and fewer empty calories consumed.

Determining the effect of fruit consumption on weight is difficult, since most studies have considered fruit and vegetables together [22, 23]; further, most studies have been conducted in adults. Although the association of fruit and vegetables consumption and weight or weight loss is inconsistent [22], the recommendation is to increase fruit and vegetable consumption in the diet to help manage weight [1, 18]. The single study that has examined apple intake and weight loss was a randomized controlled trial of overweight adult females (*n* = 411) who consumed three apples (300 g), three pears (300 g), or oat cookies (60 g) per day for 12 weeks. Groups consuming either apples or pears lost 1.21 kg, compared with the group consuming cookies that lost only 0.88 kg [24].

Ours is the only epidemiologic study that has examined apple products and weight, and the only study in children. This study showed that only total apple products and whole apples were associated with lower BMI z-scores and that consumers were less likely to be obese. Consumption of neither apple sauce nor 100 % apple juice showed any association with weight. The lack of an association between 100 % apple juice with weight is of particular import since although the majority of studies of fruit juice consumption have shown no association with weight [55, 56], one study has shown that apple juice consumption was associated with increased BMI and ponderal index in children [57]. However, that was a small regional study and the data were subsequently disputed [58]. It’s not clear why total apple products and whole apples and not apple sauce or 100 % apple juice would be inversely associated with BMI z-score, but it may be related to satiety factors, at least acutely, associated with whole fruit rather than a semi-solid food like apple sauce or a liquid like 100 % apple juice [59, 60].

The strengths of this study were that it included a large sample size with a nationally representative sample of children. The NHANES has carefully controlled protocols and screens 24-h dietary recalls confirming they are valid and complete; the NHANES also uses the multiple pass method to obtain dietary intake, which is the best dietary assessment method available for large scale epidemiologic studies. Twenty-four hour dietary recalls, used in this study do have several inherent limitations: they are memory dependent, and under- and over-reporting may occur. In proxy-assisted recalls of children, parents may know what their children consume at home [61, 62], but they may not know what their children consume outside the home, for example in school or day care [63]. Finally, cause-and-effect relationships cannot be determined from a cross-sectional study.

In conclusion, the consumption of total apple products, whole apples, apple sauce, and 100 % apple juice contributed to the fruit recommendations of children and was associated with better diet quality, and in the case of total apple products and whole apples with a decreased risk of obesity in children. Apple products should be encouraged as part of a healthy diet [64] to help children meet the recommendations for fruit.

## Abbreviations

BMI: Body Mass Index
DGA: Dietary Guidelines for Americans
HEI-2010: Healthy Eating Index-2010
NHANES: National Health and Nutrition Examination Survey
